# Supplementary material for: Comparative genomics provides new insights into the diversity, physiology, and sexuality of the only industrially exploited tremellomycete: Phaffia rhodozyma
Source: BMC Genomics. 2016 Nov 9;17:901. doi: 10.1186/s12864-016-3244-7 (PMC5103461; doi:10.1186/s12864-016-3244-7)
Supplement: Additional file 6: — List of orphan genes with links to PFAM (related to Additional file 1: Table S1). (ZIP 1428 kb) [file 12864_2016_3244_MOESM6_ESM.zip › BLAST_HTML_FTR/G02556_P.html]

BLAST Search Results


```
BLASTP 2.2.27+


Reference:
Stephen F. Altschul, Thomas L. Madden, Alejandro A. Schäffer,
Jinghui Zhang, Zheng Zhang, Webb Miller, and David J. Lipman (1997),
"Gapped BLAST and PSI-BLAST: a new generation of protein database
search programs", Nucleic Acids Res. 25:3389-3402.


Reference for
composition-based statistics:
Alejandro A. Schäffer, L. Aravind, Thomas L. Madden, Sergei
Shavirin, John L. Spouge, Yuri I. Wolf, Eugene V. Koonin, and
Stephen F. Altschul (2001), "Improving the accuracy of PSI-BLAST
protein database searches with composition-based statistics and
other refinements", Nucleic Acids Res. 29:2994-3005.


Database: nr
           71,551,133 sequences; 26,053,659,533 total letters


Query= G02556_P

Length=462
                                                                      Score     E
Sequences producing significant alignments:                          (Bits)  Value

emb|CDZ97739.1|  hypothetical protein [Xanthophyllomyces dendrorh...   897    0.0  
ref|XP_012181904.1|  predicted protein [Fibroporia radiculosa] >e...  46.6    0.051
gb|EKD46307.1|  hypothetical protein ACD_68C00042G0005, partial [...  43.9    0.22 
gb|KDQ19071.1|  hypothetical protein BOTBODRAFT_52254 [Botryobasi...  41.6    1.3  
gb|KIK54228.1|  hypothetical protein GYMLUDRAFT_264742 [Gymnopus ...  40.4    3.6  
ref|XP_013163579.1|  PREDICTED: intracellular protein transport p...  40.4    4.9  
ref|WP_015335241.1|  ribosomal protein S12 methylthiotransferase ...  39.3    9.8  


 >emb|CDZ97739.1| hypothetical protein [Xanthophyllomyces dendrorhous]
Length=472

 Score =  897 bits (2318),  Expect = 0.0, Method: Compositional matrix adjust.
 Identities = 458/472 (97%), Positives = 460/472 (97%), Gaps = 11/472 (2%)

Query  1    MSSTPSTTLLVSLLSLLAIPQPLASLCLPIHPQTPAVSRRKTRDVILRALSIHLSSPDSP  60
            MSSTPSTTLLVSLLSLLAIPQPLASLCLPIHPQTPAVSR+KTRDVILRALSIHLSSPDSP
Sbjct  1    MSSTPSTTLLVSLLSLLAIPQPLASLCLPIHPQTPAVSRQKTRDVILRALSIHLSSPDSP  60

Query  61   LHQICPKLRPLSPHEYPTPSQEKAFVGIAAGYLTELRREIISSNGLDKSWWLELKVGIRK  120
            LHQICPKLRPLSPHEYPTPSQEK+FVGIAAGYLTELRREIISSNGLDKSWWLELKVGIRK
Sbjct  61   LHQICPKLRPLSPHEYPTPSQEKSFVGIAAGYLTELRREIISSNGLDKSWWLELKVGIRK  120

Query  121  SDLSEAAGPRFDQILLALACQTVYWDILKTLQSRPELFASCAPNLSCKGQAE--------  172
            SDLSEAAGPRFDQILLALACQTVYWDILKTLQSRPELFASCAPNLSCKGQAE        
Sbjct  121  SDLSEAAGPRFDQILLALACQTVYWDILKTLQSRPELFASCAPNLSCKGQAEQRITNLFR  180

Query  173  ---TAMNEIHTLQKEPLERIKRLEERRETLLEIQALMAKPDVSEPKIRKTSSEDLRAALD  229
               TAMNEIHTLQKEPLERIKRLEERRETLLEIQALMAKPDVSEPKIRKTSSEDLRAALD
Sbjct  181  NLKTAMNEIHTLQKEPLERIKRLEERRETLLEIQALMAKPDVSEPKIRKTSSEDLRAALD  240

Query  230  DRISEWDDLIRQWFEMDGTTHSKGESNLLDPALVEMALDKAHGIPYSTAGENKAVKSSSA  289
            DRISEWDDLIRQWFEMDGTTHSKGESNLLDPALVEMALDKAHGIPYSTAGENKAVKSSSA
Sbjct  241  DRISEWDDLIRQWFEMDGTTHSKGESNLLDPALVEMALDKAHGIPYSTAGENKAVKSSSA  300

Query  290  QEMVAEFDKLETSFPQITSVVSEQLHSASGFLSKISCRLRLGPTALDEDIRFVTERVPDQ  349
            QEMVAEFDKLETSFPQITSVVSEQLHSASGFLSKISCRLRLGPTALDEDIRFVTERVPDQ
Sbjct  301  QEMVAEFDKLETSFPQITSVVSEQLHSASGFLSKISCRLRLGPTALDEDIRFVTERVPDQ  360

Query  350  TVIVSTADDLTKDIPAPSTLKLKPKAARISVSSPWKPKKSTRVTYTPLQRTSTNELRCLN  409
            TVIVSTADDLTKDIPAPSTLKLKPKAARISVSSPWKPKKSTRVTYTPLQRTSTNELRCLN
Sbjct  361  TVIVSTADDLTKDIPAPSTLKLKPKAARISVSSPWKPKKSTRVTYTPLQRTSTNELRCLN  420

Query  410  LWGLNRAPNTTSSTRSRRNDPYHHLIEHVNRTSTLSSSAERSARIQDALRRK  461
            LWGLNRAPNT SSTRSRRNDPYHHLIEHVNRTSTLSSSAERSARIQDALRRK
Sbjct  421  LWGLNRAPNTISSTRSRRNDPYHHLIEHVNRTSTLSSSAERSARIQDALRRK  472


>ref|XP_012181904.1| predicted protein [Fibroporia radiculosa]
 emb|CCM02621.1| predicted protein [Fibroporia radiculosa]
Length=734

 Score = 46.6 bits (109),  Expect = 0.051, Method: Compositional matrix adjust.
 Identities = 62/199 (31%), Positives = 85/199 (43%), Gaps = 34/199 (17%)

Query  76   YPT--PSQEKAFVGIAAGYLTELRREII----------SSNGLDK--------SWWLELK  115
            YP   PS   AF    + YL  LR  II          S N   +        SWW +  
Sbjct  72   YPCLQPSDTTAFRISLSKYLESLRHNIIHPCHAEGKKISDNEKHQGRQTIPPASWWKD--  129

Query  116  VGIRKSDLSEAAGPRFDQILLALACQTVYWDILKTLQ--SRPELFASCAPNLSCKGQAET  173
            V +RKS L E  G RF++++LAL+   ++  ILK     SRP L  S    L  +  A  
Sbjct  130  VVVRKSLLEECCGERFERLILALSTHALFKSILKLAGSPSRPSLAESSQYVLGNQSNAYI  189

Query  174  AMNEIHTLQKEPLERIKRLEERRETLLEIQALMAKPDVSEPKI----RKTSSEDLRAALD  229
                I    +   ER   +  RR   L I  L A+    EP+I       S++ L A L+
Sbjct  190  TFLNIALSARRSWERSASVLVRRAQDLVI--LRAQLSDQEPQILSRYSNFSTDRLNALLE  247

Query  230  DRISEWDDLIRQ-WFEMDG  247
               S+ +DL+R+ W   DG
Sbjct  248  ---SKHNDLLRKLWNGEDG  263


>gb|EKD46307.1| hypothetical protein ACD_68C00042G0005, partial [uncultured bacterium]
Length=268

 Score = 43.9 bits (102),  Expect = 0.22, Method: Compositional matrix adjust.
 Identities = 46/177 (26%), Positives = 71/177 (40%), Gaps = 25/177 (14%)

Query  187  ERIKRLEERRETLLEIQALMAKPDVSEPKIRKTSSEDLRAALDDRISEWDDLIRQWFEMD  246
            E +K LEE+ +TL+ I           P  R     D + +L D     DD++RQ  +  
Sbjct  41   EGLKNLEEKVDTLIVI-----------PNDRILQIIDKKTSLIDAFKAVDDVLRQGVQ--  87

Query  247  GTTHSKGESNLLD-PALVEMALDKAHGIPYSTAGENKAVKSSSAQEMVAEFDKLETSFPQ  305
                  G S+L+  P +V +       I   T      + S+S +    E  KL    P 
Sbjct  88   ------GISDLITIPGMVNVDFADVKAIMQGTGSALMGIGSASGENRAQEAAKLAIDSP-  140

Query  306  ITSVVSEQLHSASGFLSKISCRLRLGPTALDEDIRFVTERV-PDQTVIVSTADDLTK  361
               ++   +H A G L  I+    +G   +DE  R +TE + P+  VI     D  K
Sbjct  141  ---LLELSIHGAKGVLFNITGSGDMGMHEIDEAARVITESIDPNAKVIFGAVIDENK  194


>gb|KDQ19071.1| hypothetical protein BOTBODRAFT_52254 [Botryobasidium botryosum 
FD-172 SS1]
Length=342

 Score = 41.6 bits (96),  Expect = 1.3, Method: Compositional matrix adjust.
 Identities = 24/73 (33%), Positives = 40/73 (55%), Gaps = 6/73 (8%)

Query  76   YPT--PSQEKAFVGIAAGYLTELRREII---SSNGLDKSWWLELKVGIRKSDLSEAAGPR  130
            YP   P    +F   A+ Y+  LR + I   S+ G + +WW +  + +RKS L E  G R
Sbjct  80   YPCIQPVDTISFRNSASKYIESLRHQAIHPTSTKGKEGAWWWK-DIVVRKSLLEECTGER  138

Query  131  FDQILLALACQTV  143
            F+++L+AL+   +
Sbjct  139  FERVLVALSTHAI  151


>gb|KIK54228.1| hypothetical protein GYMLUDRAFT_264742 [Gymnopus luxurians FD-317 
M1]
Length=464

 Score = 40.4 bits (93),  Expect = 3.6, Method: Compositional matrix adjust.
 Identities = 31/90 (34%), Positives = 44/90 (49%), Gaps = 15/90 (17%)

Query  76   YPT--PSQEKAFVGIAAGYLTELRREIISSNGLDKSWWLELKVGIRKSDLSEAAGPRFDQ  133
            YP   PS+  AF    A YL  LR+  +  N +   WW  ++   RKS L E AG +F +
Sbjct  70   YPCLKPSETTAFRTSFARYLETLRQNSLKGNQVTSVWWRNVQA--RKSLLEECAGDKFLR  127

Query  134  ILLALACQTVYWDILKTLQS------RPEL  157
            +L+A +       IL T Q+      RP+L
Sbjct  128  LLVAFSTH-----ILHTAQAHSKVDPRPDL  152


>ref|XP_013163579.1| PREDICTED: intracellular protein transport protein USO1-like 
[Papilio xuthus]
Length=1028

 Score = 40.4 bits (93),  Expect = 4.9, Method: Compositional matrix adjust.
 Identities = 22/74 (30%), Positives = 35/74 (47%), Gaps = 6/74 (8%)

Query  300  ETSFPQITSVVSEQLHSASGFLSKISCRLRLGPTALDEDIRFVTERVP------DQTVIV  353
            E +FP     +   L+  S F S + C L  GP    E ++ VTE+ P      DQTV +
Sbjct  664  EKTFPMNEEEIQNILYFKSAFCSDVDCELVTGPITKIETVQLVTEKQPIIQETEDQTVFI  723

Query  354  STADDLTKDIPAPS  367
            +   + T ++  P+
Sbjct  724  TLEKNCTDELLKPN  737


>ref|WP_015335241.1| ribosomal protein S12 methylthiotransferase RimO [Desulfovibrio 
hydrothermalis]
 emb|CCO22633.1| Ribosomal protein S12 methylthiotransferase RimO [Desulfovibrio 
hydrothermalis AM13 = DSM 14728]
Length=437

 Score = 39.3 bits (90),  Expect = 9.8, Method: Compositional matrix adjust.
 Identities = 30/86 (35%), Positives = 47/86 (55%), Gaps = 13/86 (15%)

Query  193  EERRETLLEIQALMAKPDVSEPKIRKTSSEDLRAALDDRISEWDDLI--RQWF---EMDG  247
            EERR  L+EIQ+ +++ D+ E KI +T    ++  +++   EW  L   R WF   E+DG
Sbjct  347  EERRLELMEIQSAISR-DILEEKIGET----IQVLIEEPNEEWPGLFNGRVWFQAPEVDG  401

Query  248  TTH---SKGESNLLDPALVEMALDKA  270
             T+    +GES L    L+E  +D  
Sbjct  402  ITYISAPEGESELKPGMLIEAEIDNV  427


Lambda      K        H        a         alpha
   0.316    0.130    0.376    0.792     4.96 

Gapped
Lambda      K        H        a         alpha    sigma
   0.267   0.0410    0.140     1.90     42.6     43.6 

Effective search space used: 4630713795708


  Database: nr
    Posted date:  Sep 23, 2015 12:05 AM
  Number of letters in database: 26,053,659,533
  Number of sequences in database:  71,551,133


Matrix: BLOSUM62
Gap Penalties: Existence: 11, Extension: 1
Neighboring words threshold: 11
Window for multiple hits: 40
```
